# Preparation of silver nanowires with controlled parameters for conductive transparent electrodes

**DOI:** 10.1038/s41598-024-70789-6

**Published:** 2024-09-09

**Authors:** Ahmed Abdel Salam, Shaker Ebrahim, Moataz Soliman, Azza Shokry

**Affiliations:** Department of Materials Science, Institute of Graduate Studies and Research, Alexandria University, P.O. Box 832, Alexandria, Egypt

**Keywords:** Silver nanowires, Polyvinylpyrrolidone, Control agent, Polyol, Transparent electrode, Chemistry, Materials science, Nanoscience and technology

## Abstract

Silver nanowires (AgNWs) have excellent flexibility, unique optical transmittance and high conductivity. The polyol process is appropriate for preparing AgNWs due to its simplicity, effectiveness, low cost, and high yield. This work aims to investigate the effect of preparation parameters of the polyol process on the silver nanowires properties. The parameters include the controlling agent, molecular weight of the polyvinylpyrrolidone (PVP), the temperature, and the reducing agent. The amount of silver nanoparticles formed during preparation was used to determine the optimum preparation conditions. The transmission electron microscope (TEM) images showed minimal amount of Ag nanoparticles when using mixed molecular weight of PVP-40K, and PVP-1.3M at 150 °C with the assistance of copper chloride as a controlling agent. The prepared AgNWs had an average length of 3.7 µm and aspect ratio of 15.3. The fabricated electrodes were characterized using a scanning electron microscope (SEM) and four probe resistivity measurements. The electrical measurement of the AgNWs electrodes indicated that the surfactant thickness is a critical parameter in having low sheet resistance electrodes. Also, the optical transmission was affected by the amount of nanoparticles. The prepared electrode with high concentration of AgNWs and a minimal amount of nanoparticles exhibited 80% optical transmission.

## Introduction

Transparent conducting electrodes (TCEs) play an important role in organic optoelectronic devices, such as photovoltaic cells, light-emitting diodes, and displays^1^. Two main factors are essential for TCE’s, namely optical transmission, and sheet resistance. Many studies explored various materials for high transmission and low sheet resistance. Flexible electronics, in particular rollable electronics, such as flexible solar cell panels, screen mobile phones, and curved display devices will be susceptible to various mechanical deformations, such as bending, stretching, and twisting. The transparent electrodes used in the mainstream devices in the market are generally transparent conductive oxide (TCO)^2^. Indium tin oxide (ITO) is in the growing market since it is required for many displays^3^, touch panels^4^, solid-state lighting^5^, photovoltaic cells^6^, and e-paper^7^. ITO is incompatible with bending cycles and its efficiency is decreased during and after bending^1^. Therefore, the mechanical property besides transmission and conductivity should take into consideration in the transparent conducting electrode^8^. As a result, numerous unique electrodes have been developed relatively as the electrode’s flexibility is a major property in TCEs. Various indium-free transparent electrodes such as poly(3,4-ethylenedioxythiophene) polystyrene sulfonate (PEDOT:PSS)^9^, graphene electrodes^10^, oxide electrodes^11^, and AgNWs^12^ have been extensively investigated as replacements for the ITO electrodes. Several techniques for AgNWs preparation emerged to obtain a high aspect ratio of Ag NWs^13–16^. The polyol method has gained considerable interest since it is simple, scalable, and shows much control over nanowire morphology^17^. Yamamoto et al.^18^ evaluated the effect of NaCl, CuCl_2_, and NaBr as control agents on the aspect ratio of AgNWs and found that the lowest aspect ratio formed using NaBr compared to chloride ions. Using copper chloride increased the purity of AgNWs by reducing the percentage of Ag nanoparticles to that AgNWs. Alqanoo et al.^19^ prepared AgBr_x_Cl_x-1_ crystals by mixing two different types of halides (Cl and Br) at a fixed ratio. The results confirmed that the high aspect ratio of AgNWs was strongly secured by AgCl rather than BrCl. Chen et al.^20^ discussed the UV–Vis spectra at different stages of the formation of AgNWs prepared by the polyol process. Silver peaks were not observed when the reaction temperature was below 140 °C. As the temperature increased to 150 °C, two weak peaks appeared around 359 and 370 nm indicating the formation of small AgNWs and large AgClBr_2_ crystals. At 160 °C, Chen et al. confirmed the existence of a high amount of AgNWs and they discussed the reaction’s yield at 160 °C and different reaction time. Fahad et al.^21^ investigated the effect of reaction temperature on the silvers’ morphology using various characterization techniques. They observed that at high temperature severe coagulation of the silver atoms were formed to produce large silver nanoparticles. It is not recommended to increase the reaction temperature up to 180 ֯C, as the adsorption potential of the PVP decreases on the silver’s surface due to the lower melting point of multiple- twinned particles (MTPs) than bulk material^22^. This will cause the deposition to be randomly distributed on the silver’s surface^23^. Lau et al.^24^ studied the function of PVP chain length and they found that the high molecular weight of PVP is necessary to produce AgNWs with a smaller diameter and longer wires, which are required for the transparent conductive electrodes. Sonntag et al.^25^ investigated the influence of PVP Molecular weight on the conductivity of AgNWs network. They observed that as the molecular weight moved from 40 to 55K, the AgNWs diameter is decreased, meanwhile, the diameter is decreased as the molecular weight goes higher from 360K to 1.3M. The PVP chain length is decisive not only for governing the AgNWs growth but also during the nucleation of Ag nanoparticles. Li et al.^26^ argued that AgNWs cannot be formed using PVP with molecular weight (Mw) below 8000, and AgNWs cannot be formed till the PVP MW runs to 29,000. Li et al. demonstrated that the reaction’s time diminishesd as the PVP molecular weight becomes 1,300,000. The AgNWs were formed in the early stage of the reaction at 10 min after adding the precursor. The higher viscosity of the solution may reduce the nucleation rate^27,28^.

This work aims to determine the optimum conditions for synthesizing AgNWs for transparent conductive electrodes using polyol process. We optimized the control agents, molecular weights of PVP, reaction temperatures, and reducing agents to determine the proper conditions for a high aspect ratio of AgNWs with high optical transmission. Several characterization techniques had been used to elucidate the morphological, structural, and optical characteristics of AgNWs, namely UV–Visible spectrophotometer (UV–Vis), Fourier transform Infrared spectroscopy (FT-IR), TEM, and X-ray diffraction (XRD). The prepared AgNWs were utilized to prepare transparent conductive electrodes using a drop-casting approach. The prepared electrodes also were characterized to study the effect of preparation parameters on their performance.

## Experimental section

### Chemicals

Silver nitrate (99.8%) was obtained from DOP Organic Kimya SAN.VE, copper chloride (CuCl_2_) (93%) was obtained from May & Baker LTD, England. Hydrochloric acid (37%) and ethanol (99.5%) were purchased from El Nasr Pharmaceuticals Chemicals (Egypt). PVP powder- 40K and diethylene glycol 99% (DEG) were received from Alfa Aesar. PVP powder-1,3M was supplied from ACROS Organics. Ethylene glycol 99% (EG) was obtained from Piochem. Sodium hydroxide (98%) and potassium hydroxide (85%) were obtained from Gateway Company.

### Preparation of AgNWs

This study performs Korte’s method to synthesize AgNWs^29^. Ten milliliters of solvent (EG/DEG) were heated in a two-neck flask at 150 °C for 1 h under magnetic stirring (400 rpm). Subsequently, 80 μL of 4 Mm of controlling agent (CuCl_2_.2H_2_O or HCl) solution was injected into the flask. After waiting for 15 min to ensure that all chloride ions were decomposed, 3 mL of 0.147 M of PVP with (40K/1.3M) Mw solution was introduced to the above solution, followed by the addition of 3 mL of 0.094 M AgNO_3_ and monitoring the color changes from yellow to grey after reaction completion within 1.5 h. The resulting ink was collected by centrifuging process (Focus serial No: 1107, Spain) at 6000 rpm for 20 min. The ink was washed three times using ethanol and twice with purified water to remove the nanoparticles and reduce the thickness of PVP.

### Preparation AgNWs transparent electrode

Glass substrates were cut into small pieces of the same size, cleaned ultrasonically using Hellmanex, purified water, and ethanol for 20 min for each solvent, and dried for 24 h to remove any solvent residues. Then, 80 µL AgNWs is evenly drop-casted on the glass substrates at 130 °C using a micro-pipette. The resulting Ag NWs electrodes were dried at 100 °C for 12 h. Finally, Ag NWs electrodes were immersed in 1 M KOH solution (alkaline treatment) to remove the PVP layer and dried at 100 °C for 24 h.

### Characterization techniques

The optical characteristic peaks of AgNWs and the transparency of the conductive electrodes were examined by UV–Vis spectrophotometer (Evolution 300, Thermo Fischer Scientific) at wavelength range of 300–750 nm. The functional groups of AgNWs electrodes were examined using FTIR and the spectrum was recorded in the range of 4000–400 cm^−1^ using a spectrum BX 11- LX 18–5255 Perkin Elmer. The crystallinity of AgNWs ink was investigated using X-ray powder diffraction (XRD-D2 phaser, Bruker, Germany). The ink was dropped casting on the thin glass, followed by heating to remove any water molecules from the glass surface. The morphology and aspect ratio of AgNWs were explored using transmission electron microscope (TEM) (Gatan digital micrograph software, JEOL. JEM-2100F) at 200 kV. A powder of AgNWs was dispersed in ethanol and sonicated for 15 min. 5 μL of the dispersed solution was dropped on a carbon coated copper grid. The topography and shape of the final electrode were examined by scanning electron microscope (SEM) (JEOL JSM 6010-LV). The sample was stacked into a double-faced carbon tab, then coated with Pt–Pd counting source using JEOL auto fine counter. The sheet resistance of the thin layer was determined in ohm per square using the SIGNATONE four point-probe device.

## Results and discussion

Different control agents are utilized during the polyol process to control the reduction rate of silver and form a coordination bond to prohibit silver atoms from the accumulation onto (100) facet. The common anion used was chloride ions which can produce silver chloride and control the nucleation process of AgNWs^30^. 

### Effect of controlling agents

Different control agents are utilized in the polyol process, to control the reduction rate of silver and make a coordination bond to prohibit any silver atoms from accumulation. The chloride ions support the multi-twinned structures to keep all particles in small size and reducing the reaction rate through competing with Ag^+^ via coordination bond^31^. The current study investigates the impact of hydrochloric acid and copper chloride as control agents in the polyol process using different molecular weights of PVP.

### Using hydrochloric acid as a controlling agent

**Fig. 1:**
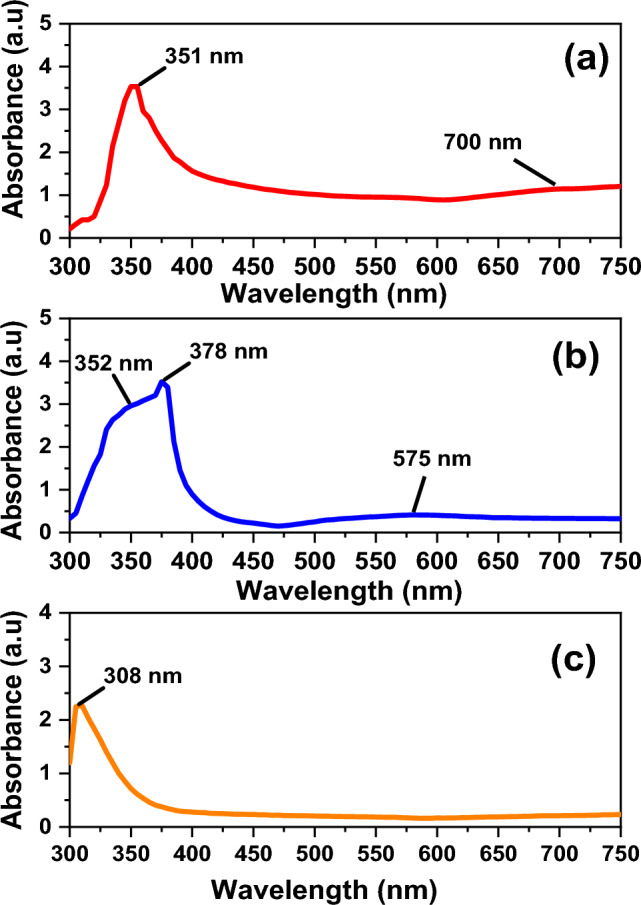
UV–Vis spectra of AgNWs using HCl with different molecular weights of PVP (**a**) PVP-40K, (**b**) PVP-1.3M, (**c**) volume ratio 1:1 of PVP-40K and PVP-1.3M.

Figure 1a shows broadness and high intensity of the shoulder that may be due to an increase in the AgNWs diameter^32^. The broad shoulder emerged at 700 nm shows that AgCl seed size is smaller^33^. This leads to longer length of AgNWs. Whilst using PVP-1.3M with HCl, the shoulder peak surfaced is blue-shifted to 575 nm, this indicates a larger AgCl seeds and shorter length of AgNWs is obtained^34^. PVP-1.3M enhances the half peak width of the surface plasmon, indicating the formation of AgNWs, as shown in Fig. 1b^35,36^. Using mixed capping agents, only one wide peak is observed at 308 nm in Fig. 1c, which may correspond to the surface plasmon absorption band of small nanoparticles^37^. The results of UV–Vis spectrum shows that the AgNWs could be obtained using PVP-40K and PVP 1.3-M, while the findings in Fig. 1c may demonstrate that the AgNWs could not be attained using HCl.

**Fig. 2:**
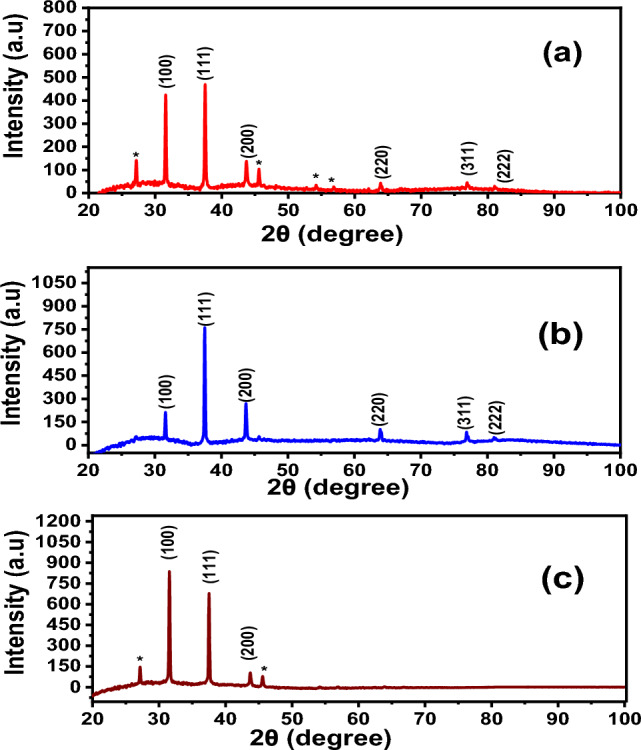
XRD patterns of a synthesized using HCl with different molecular weights of PVP (**a**) PVP-40K, (**b**) PVP-1.3M (**c**) volume ratio 1:1 of PVP-40K and PVP-1.3M.

The synthesis of AgNWs is confirmed by the XRD patterns. The peaks at 37.63°, 43.92°, 64.83°, and 81.20° are indexed to (111), (200), (220), and (222), respectively^37^, as presented in Fig. 2. The peak of (111) facet is attributed to the longitudinal of AgNWs, while (100) facets indicate poor capping agent of PVP on AgNWs surface of the (100) facets^37^. XRD of AgNWs prepared by HCl with PVP-40K in Fig. 2a, shows several additional diffraction peaks (*) corresponding to the existence of AgCl in the final yield^34^. The ratio between the planes (111) and (200) is low, indicating low existence of AgNWs^38,39^. The lattice constant using PVP-40K is 4.12 Å and reduces to 4.11 Å using PVP-1.3M, which matches the reference JCPDS file 04-0783^40^. The intensity ratio between the planes (111/200) is 2.83 with appearance of (100) plane for samples prepared using PVP-1.3M, as shown in Fig. 2b. Using mixed of PVP in Fig. 2c is inappropriate, since the plane (100) shows higher intensity than that of plane (111), which means that the majority of AgNWs are not covered with PVP^41^, as well as the growth direction is low in the plane (111).

FT-IR spectra are utilized to study the interaction between the pyrrolidone ring and the silver surface^42^. The intensity of the coordination interaction between PVP and Ag^+^ could be evaluated by the occurrence of band shifts in the FT-IR spectrum^43^. The observed band at 2970 cm^-1^ is assigned to the symmetric stretching vibration of CH_2_ in the skeletal chain of PVP, while the distinct peak at 3000–3500 cm^-1^ is assigned to the stretching vibration of the -OH group^44^. The shown bands at 1625 cm^-1^ and 1237.1 cm^-1^ are attributed to C = O stretching vibration from PVP and in-plane vibration of the C-N group, respectively^45^. In Fig. 1S, the characteristic peaks are well-defined related to PVP molecules and their adsorption interaction with silver’s surface^46^. The two distinct peaks that attributed to PVP at 3456.41 cm^-1^ and 1635.62 cm^−1^ have a blue shift, revealing that the reaction using PVP-40K has high amount of PVP^47^, as seen in Fig. 1Sa. In contrast, samples prepared using the high molecular weight of capping agent in Fig. 1Sb, the peak becomes narrow with low transmittance, indicating high amount of PVP in the final yield. When mixed PVP is utilized, high transmittance of stretching vibration bands at 1639.48 cm^−1^ and 3460.27 cm^−1^ which refers to low amount of PVP in the final yield, as shown in Fig. 1Sc.

**Fig. 3:**
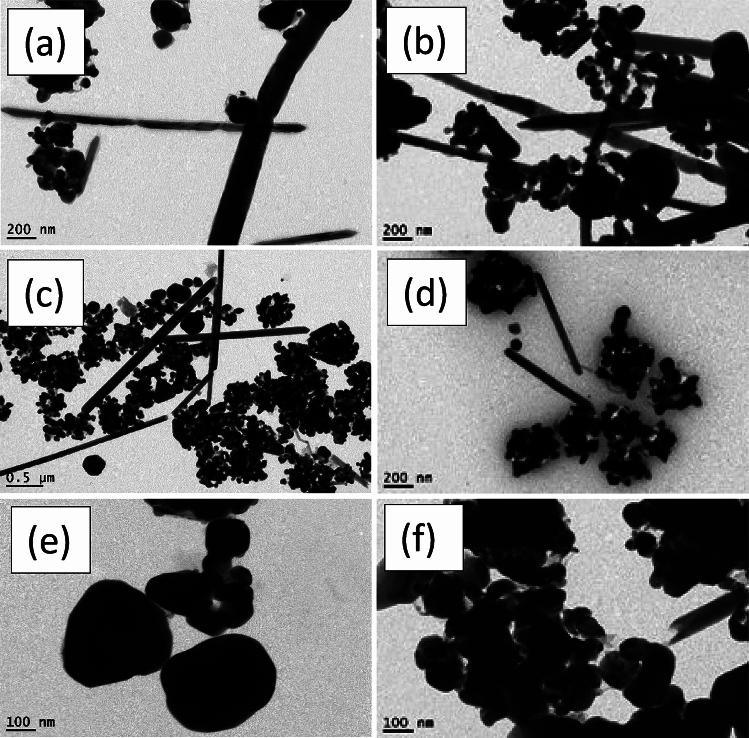
TEM images of synthesized AgNWs using HCl with different molecular weight of PVP (**a**,**b**) PVP-40K, (**c**,**d**) PVP-1.3M, (**e**,**f**) volume ratio 1:1 of PVP-40K and PVP-1.3M.

Figure 3 illustrates the morphologies of the final yield. AgNWs samples prepared using HCl contained high amount of silver nanoparticles. The length and diameter of AgNWs prepared using PVP-40K were 1.5 µm and 168 nm, respectively, and having a low aspect ratio of 9, as shown in Fig. 3a,b. For AgNWs prepared with PVP-1.3M as shown in Fig. 3c,d, the length of AgNWs is increased to 1.9​ µm and the diameter is decreased to 136 nm and the aspect ratio is raised to 14, which indicate the necessity of using high molecular weight of PVP to synthesize high aspect ratio of AgNWs. High Mw of PVP slows the reduction rate of Ag nanoparticles and this reflected on the growth of AgNWs^21^. Moreover, longer chains of PVP create a steric hindrance effect. This means blocking (100) facet of nanowires, accordingly, promoting the formation of elongated nanowires. Using mixed PVP, most of the silver nanoparticles formed as depicted in Fig. 3e,f are accumulated together with the emergence of PVP adsorbed on (100) facet. This is evidence that the crystalline surface contains active (100) facet in the final yield^39^.

### Using copper chloride as a controlling agent

**Fig. 4:**
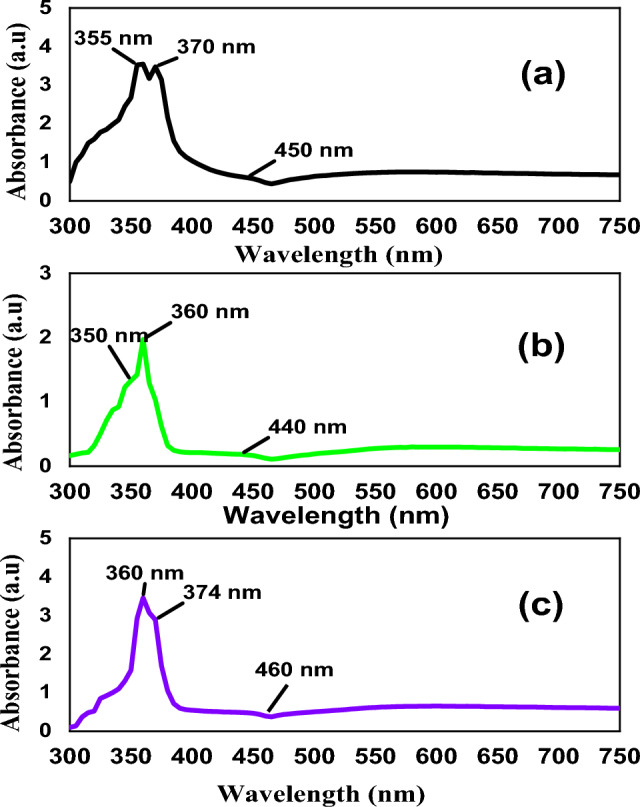
UV–Vis spectra of AgNWs prepared using CuCl_2_ and different molecular weights of PVP (**a**) PVP-40K, (**b**) PVP-1.3M, (**c**) volume ratio 1:1 of PVP-40K and PVP-1.3M.

Using cations have two oxidation states can prohibit the oxidative etching on the silver’s surface^31^, and form long and thin AgNWs in the final yield. Figure 4 illustrates the UV–Vis spectra of AgNWs synthesized by copper chloride at different molecular weights of PVP. Three distinguishable absorption peaks of AgNWs appeared in the spectra, with high absorption as shown in Fig. 4a. The absorption peak at 360 nm of transverse plasmon mode of AgNWs in PVP-1,3M is blue shifted due to high aspect ratio of AgNWs in this reaction^36^. Also, the distinguished peak at 450 nm which corresponds to silver nanoparticles is blue shifted to 440 nm that may be due to substantial prevalence of nanoparticles in the final yield, as shown in Fig. 4b^32^. Using mixed PVP in Fig. 4c, the distinctive peaks is attributed to the surface plasmon of AgNWs are narrow, confirming that the AgNWs formed successfully. While the distinctive peak at 450 nm attributed to silver nanoparticles has lower absorbance, indicating that the prepared silver nanowires are pure with minimal amount of nanoparticles^48^.

**Fig. 5:**
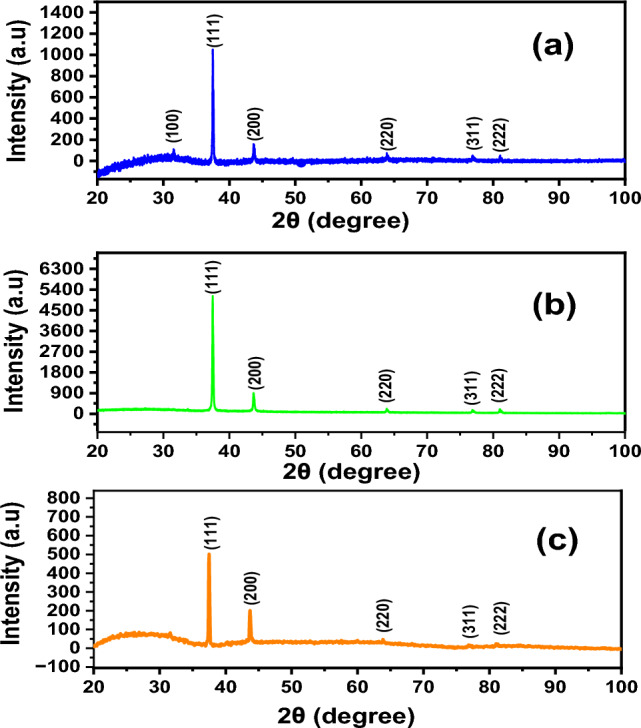
XRD patterns of AgNWs prepared using copper chloride with different molecular weights of PVP (**a**) PVP-40 K, (**b**) PVP-1.3 M, (**c**) volume ratio 1:1 of PVP-40K and PVP-1.3M.

For preparing AgNWs using CuCl_2_ and PV-40 K, XRD peaks are attributed to silver’s crystal lattice displayed alongside (100) facet with 6.6 aspect ratio between (111) and (200) planes of AgNWs and a lattice constant of 4.1240 Å, as shown in Fig. 5a. However, using PVP-1.3 M with copper chloride, the prepared AgNWs show a high aspect ratio between (111) and (200), equal to 5.6, which refers to the high amount of AgNWs yield, as shown as in Fig. 5b. XRD patterns show the advantages of using mixed PVP, because the aspect ratio is found to be 2.5 using CuCl_2_ as a controlling agent (Fig. 5c), which matches with the reference JCPDS file 04–0783^46^. All lattice constants are close to the theoretical values of AgNWs, as shown in Table 1^49^. XRD results confirmed that using mixed PVP is effective in synthesizing AgNWs.

**Table 1:** Lattice constants calculated for different crystalline AgNWs prepared using polyol process with different controlling agents and capping agent having different molecular weights.

Sample	CuCl_2_ /PVP-40 K	HCl/PVP-40 K	CuCl_2_ /PVP-1.3 M	HCl/PVP-1.3 M	CuCl_2_/PVP-1:1	HCl/PVP-1:1
Lattice constant (Å)	4.1240	4.1249	1.1247	4.1051	4.1266	4.1017
Theoretical value of JCPDS file 04–0783 (Å)				4.0892		

For FTIR spectra for AgNWs prepared using CuCl_2_ and PVP-40 K, all distinct peaks.

at 3458.34 cm^−1^, and 1639.48 cm^−1^ are well-defined to the stretching vibration of O–H and C=O, respectively. The high transmission emphasises low existence of PVP-40 K alongside the silver’s surface, as shown in Fig. 2Sa. FTIR spectrum of AgNWs prepared using PVP-1.3 M in Fig. 2Sb, the peak attributed to the stretching vibration of the -OH group at 3448.69 cm^−1^ is shortened due to the formation of AgO molecules^48^. The bands corresponding to C=O stretching vibration and O–H in Fig. 2Sb occurred at 1660.7 and 3448.69 cm^−1^, respectively, became wider confirming low existence or dissolution of PVP. On the other hand, using mixed PVP, FTIR spectrum shown in Fig. 2Sc indicates sharp peaks of C=O at 1643.34 cm^−1^ and 3450.62 cm^−1^ that attributed to stretching vibration of O–H in pyrrolidone ring with low transmittance, and confirming PVP is strongly adsorbed onto the silver’s surface^46^.

**Fig. 6:**
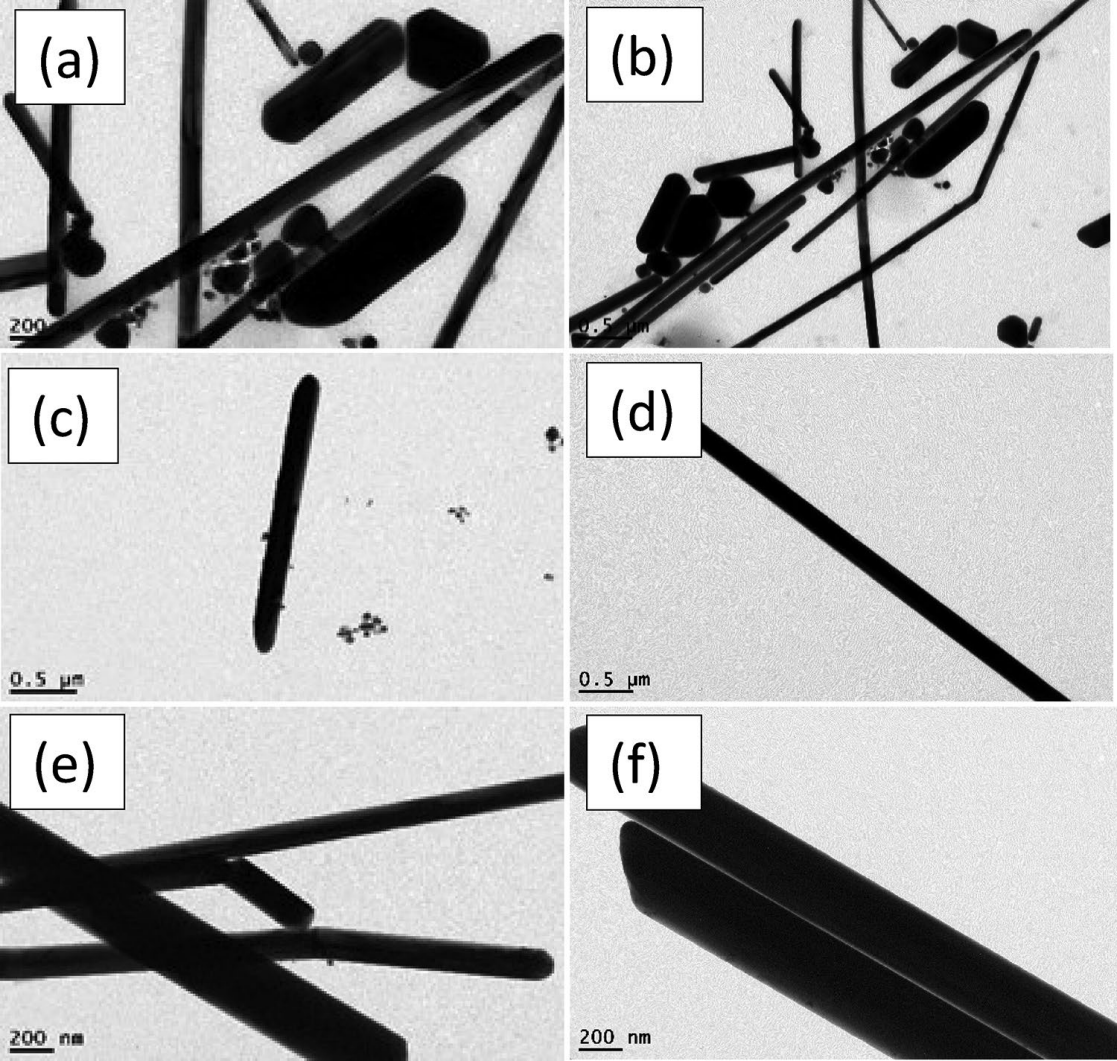
TEM images of AgNWs synthesized using CuCl_2_ with different molecular weights of PVP (**a**,**b**) PVP-40 K, (**c**,**d**) PVP-1.3 M, (**e**,**f**) volume ratio 1:1 of PVP-40K and PVP-1.3M.

Figure 6 displays the morphologies of AgNWs synthesized using CuCl_2_ and different molecular weights of PVP. When PVP-40 K employed with copper chloride, AgNWs are uniform and have high degree of purity, with length of 2.275 µm, diameter of 120.57 nm, and aspect ratio of 19, as shown in Fig. 6a,b. Due to the dissolution of PVP-1.3 M, some silver nanoparticles are adsorbed onto the silver nanowires surface onto (100) facet, as shown in Fig. 6c,d. Therefore, the estimated diameter and length of AgNWs are enlarged to 259 nm and 3.3 µm, respectively and the aspect ratio is decreased to 12.7. Figure 6e,f show long, thin, and pure nanowires synthesizing using mixed PVP, and the average diameter and length of AgNWs are 242.96 nm and 3.8 µm, respectively, with aspect ratio of 15.6. In addition, using mixed PVP forms a 10 nm thick PVP layer on the (100) facet.

Table 2 indicates higher values of aspect ratio when copper chloride is utilized. Due to the dissolution of PVP-1.3 M, more nanoparticles accumulate on AgNWs surface and widen the diameter further. Therefore, the preparation conditions of mixed molecular weights of PVP and copper chloride are chosen to prepare AgNWs and to study the effect of temperature and reducing agent. Our findings on the length, diameter and aspect ratio of AgNWs are consistent with previous reports. lau et al.^24^ stated that increasing the PVP molecular weights from 55 K to 1.3 M promote the growth of AgNWs in (100) direction. Moreover, using hydrothermal of biomass and active ingredients, Li et al.^26^ synthesized AgNWs with diameter of 77 nm and length of 10 µm. The authors argued that there is a positive correlation between PVP molecular weights and length of AgNWs.

**Table. 2.** The lengths, diameters and aspect ratio of prepared AgNWs prepared with different reagents**.**

Sample	CuCl_2_/PVP-40 K	HCl/PVP-40 K	CuCl_2_/PVP-1.3 M	HCl/PVP-1.3 M	CuCl_2_/PVP-1:1
Length (µm)	2.275	1.5	3.3	1.9	3.8
Diameter (nm)	120.57	168	259	136	242.96
Aspect ratio	19	9	12.7	14	15.6

### Effect of synthesizing temperature

A sufficient temperature is required to offset the deficiency of thermal energy for formation of specific facets of AgNWs. Thus, studying temperature is a crucial factor to optimize the required temperature to produce pure with high aspect ratio of silver nanowires. The current work studied the temperature, above and below 150 °C by 20 °C, on the final yield and silver atom transformations in the polyol reaction. 

Figure 3S shows the optical absorbance of AgNWs synthesized at different temperatures, namely 130 °C, 150 °C and 170 °C. At 130 °C. It is observed that Localised Surface Plasmon Resonance (LSPR) peak of AgNWs is not detected, and only a small peak at 465 nm of silver nanoparticles is noted, as shown in Fig. 3Sa. This revealing that the temperature is not sufficient to accelerate the reduction process, and silver atoms are started to accumulate through Ostwald ripening process to form nanoparticles^50,51^. Meanwhile increasing the reaction’s temperature to 150 °C, the prepared AgNWs have a slightly smaller half-peak width of LSPR, as shown in Fig. 3Sb, indicating a decrease in AgNWs diameter^36^. Moreover, at 170 °C in Fig. 3Sc, AgNWs LSPR peaks have red-shifted to a higher wavelength, revealing an increase of AgNWs diameter at high temperature^52,53^.

**Fig. 7:**
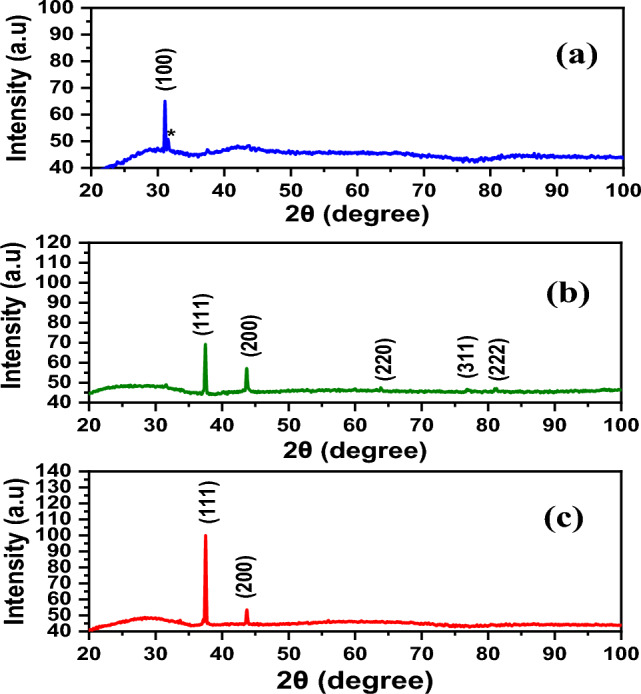
XRD patterns of AgNWs synthesized using CuCl_2_ and mixed PVP at different temperatures (**a**) 130 °C, (**b**) 150 °C, (**c**) 170 °C. * is attributed to AgCl.

Figure 7 depicts XRD of AgNWs prepared using CuCl_2_ and mixed PVP at different temperatures. At 130 °C, the thermal energy is insufficient to form (111) facet and contain only (100) facet, and the final yield missed any dominant of AgNWs peaks^54^. In Fig. 7b, when the temperature is increased to 150 °C, XRD pattern shows that all silver facets predominate in the final yield with an intensity ratio of (111/200) facet 1.88 and lattice constant of 4.1266 Å. At 170 °C, high thermal energy is available to form AgNWs, and excessive (111) facets predominate in the final yield, as shown in Fig. 7c^55^.

The FT-IR spectra in Fig. 4S demonstrate PVP-AgNWs interaction at different temperatures. In comparison to high temperature, the transmittance at 130 °C recording around 65% (Fig. 4Sa). This may be due to lack of enough thermal energy to make (111) facet; as a result, the PVP failed to be adsorbed onto silver’s facets, and dissolve in the washing process of the final yield. ​The spectrum in Fig. 4Sb shows a blue-shifted peak that is attributed to C=O of PVP to 1651.05 cm^−1^, while in Fig. 4Sc the peak has red-shifted to 1647.19 cm^−1^, implying a strong adsorption of PVP onto the silver’s surface^46^. 

**Fig. 8:**
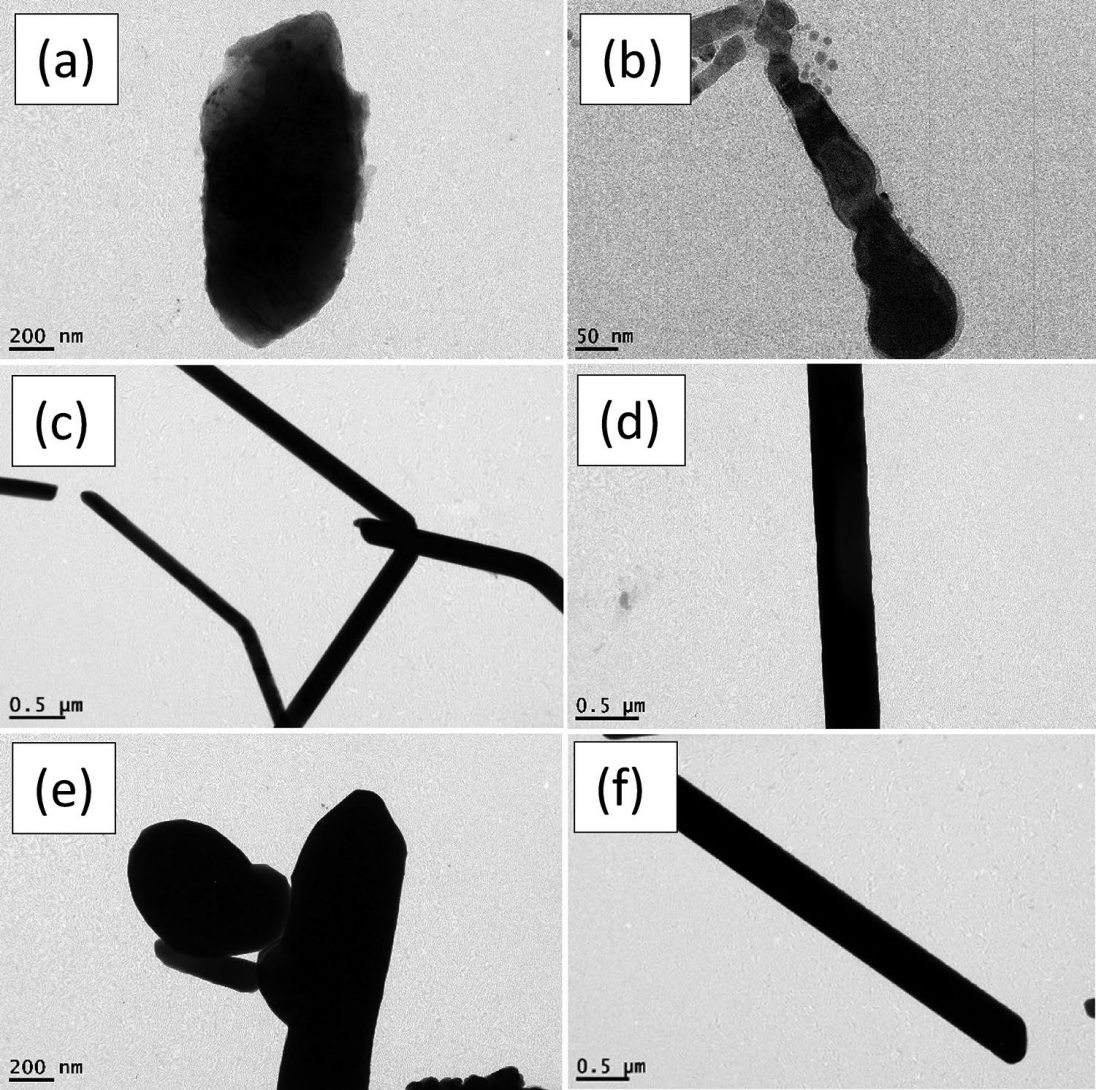
TEM images of AgNWs synthesized using CuCl_2_ and mixed PVP at different temperatures (**a**,**b**) 130 °C, (**c**,**d**) 150 °C, (**e**,**f**) 170 °C.

Figure 8 discusses the morphology of AgNWs formed at different temperatures using CuCl_2_ and mixed PVP. Most of the silver atoms synthesized at 130 °C are nanoparticles. There is not enough thermal energy to oxidize the ethylene glycol to glycolaldehyde in which most of the silver cation remained in the solution as well as deficiency of thermal energy becomes clear in TEM in Fig. 8a,b^55^. As shown in Fig. 8c,d, long and thin nanowires are synthesized at 150 °C with a high aspect ratio of 15.6 which indicates the whole ethylene glycol was converted to glycolaldehyde to assist in the reduction process^54,56^. It is found that above the critical temperature, the aspect ratio is decreased with increasing the number of silver nanoparticles. Excessive available thermal energy in the solution becomes useless, and silver atoms start to accumulate to each other in a random way with less tendency to form uniform nanowires at 170 °C in Fig. 8e,f^52^. Accordingly, the optimum temperature in the reaction is 150 °C to have AgNWs with high aspect ratio.

### Effect of reducing agents

To investigate the influence of reducing agent on the final yield, the shape and average dimensions of silver nanowires are compared using diethylene glycol. The shown UV–Vis spectrum in Fig. 5S indicates two different patterns of AgNWs synthesized with different reducing agents. The characteristic peaks of AgNWs are narrow with sharp absorption in the case of using ethylene glycol, as seen in Fig. 5Sa, compared to wider and weak peaks that appeared when using diethylene glycol, as shown in Fig. 5Sb. All peaks in Fig. 5Sb are blue-shifted, which confirms the long non-uniformity of AgNWs, while wider absorption peaks for surface plasmon response indicate an increasing diameter of AgNWs^34^.

**Fig. 9:**
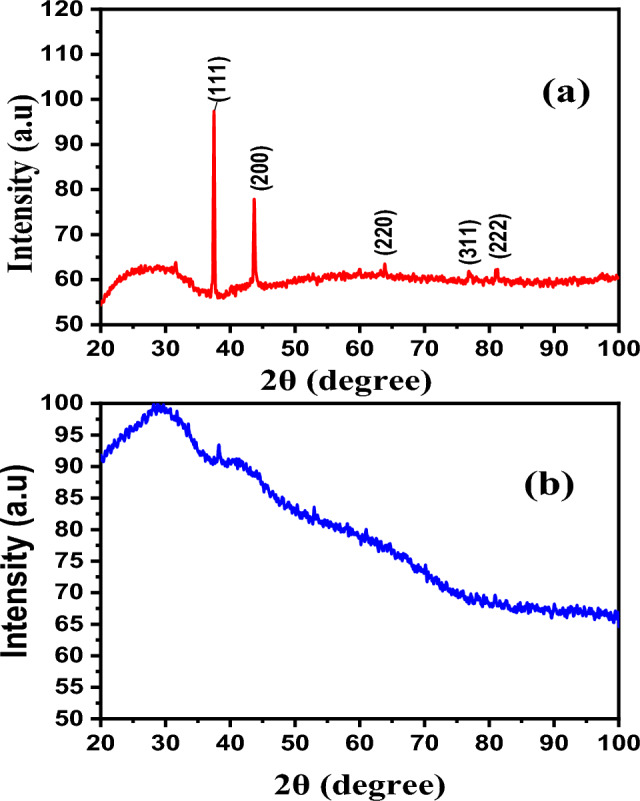
XRD patterns of AgNWs prepared using CuCl_2_ and mixed PVP with different reducing agents (**a**) ethylene glycol, (**b**) diethylene glycol.

The XRD patterns in Fig. 9 show formation of pure crystalline AgNWs prepared using ethylene glycol with an intensity ratio of (111/200) facets of 1.88 and lattice constant of 4.13 Å, as shown in Fig. 9a. These results agree with the reported data (2.5 and 4.0892 Å), respectively, from the JCPDS file 04-0783^57^. Using diethylene glycol as a reducing agent, non-crystalline NWs were formed, as shown in Fig. 9b.

**Fig. 10:**
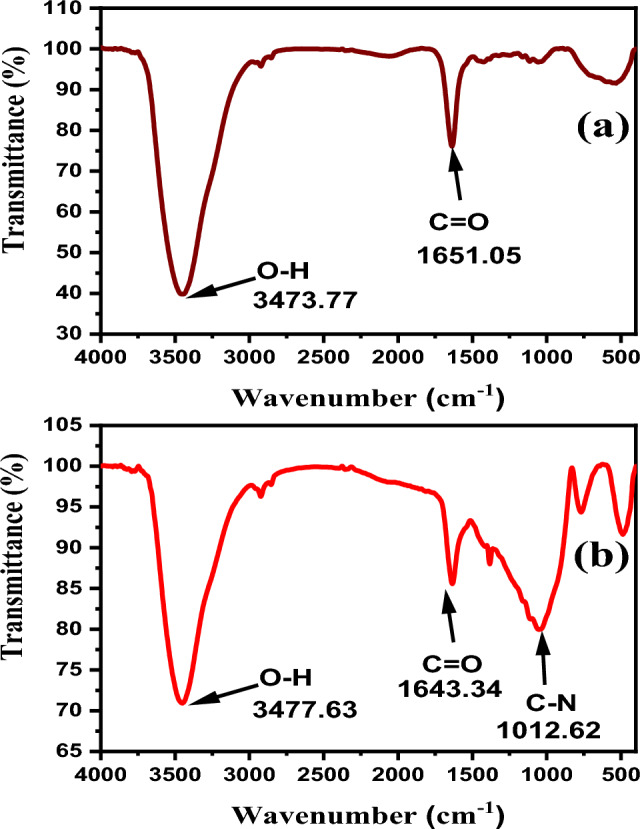
FT-IR spectra of AgNWs synthesized using CuCl_2_ and mixed PVP with different reducing agents (**a**) ethylene glycol. (**b**) diethylene glycol.

Figure 10 displays the differences in FT-IR spectra of prepared AgNWs using different reducing agents. In the case of ethylene glycol in Fig. 10a, sharp peaks of stretching vibration of C=O and O–H in PVP skeletal ring, revealing that PVP is adsorbed uniformly on the silver’s surface^46^. Figure 10b demonstrates intense peaks below 1400 cm^−1^ that mentioned a significant interaction between C-N and silver surface and NO_3_^–1^^58^. FT-IR confirms the hypothesis that silver nitrate molecules and PVP accumulate to form non-crystalline nanowires.

**Fig. 11:**
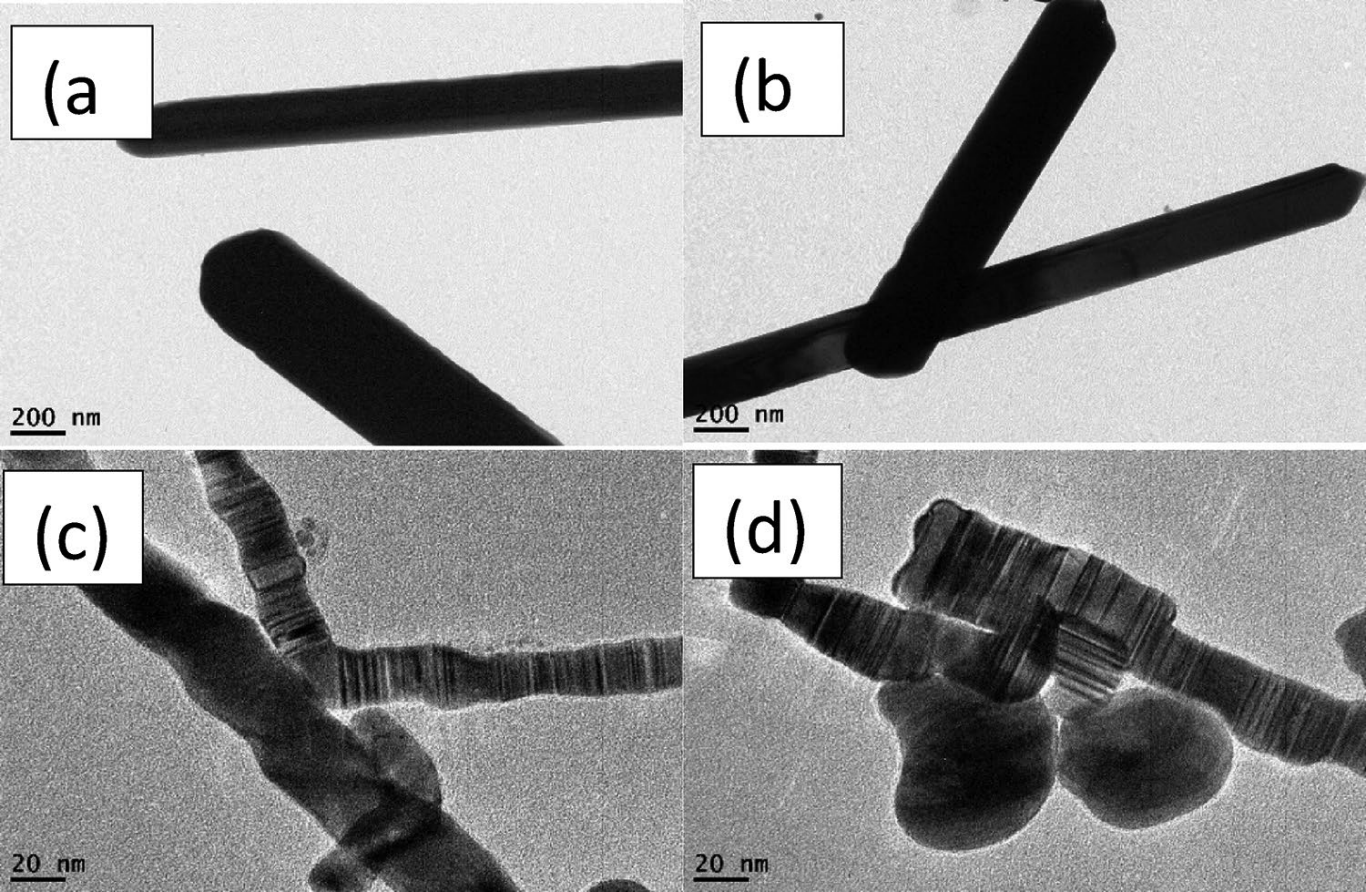
TEM images of AgNWs AgNWs synthesized using CuCl_2_ and mixed PVP with different reducing agents (**a**,**b**) ethylene glycol (**c**,**d**) diethylene glycol.

Figure 11 displays the morphology of AgNWs formed by two different reducing agents. High yield of AgNWs are formed using ethylene glycol as a reducing agent to reduce silver cations, as shown in Fig. 11a,b. TEM images in Fig. 11c,d illustrate the formation of non-crystalline silver NWs as well as nanoparticles using diethylene glycol. These particles are formed because diethylene glycol has no ability to reduce silver cations into silver atoms, which leads to the accumulation of the silver nitrate and PVP molecules into wire shapes.

### Preparation of transparent conducting electrode

The transparent conducting electrodes in Fig. 6S are prepared using the drop-casting method and subjected to the same treatment to purify the silver's surface of any organic content. As the AgNWs concentrations increase on the coated substrate, the transmittance is dropped, and sheet resistance is decreased, meanwhile existence of nanoparticles reduces the electrical properties of the electrode^59–61^.

**Fig. 12:**
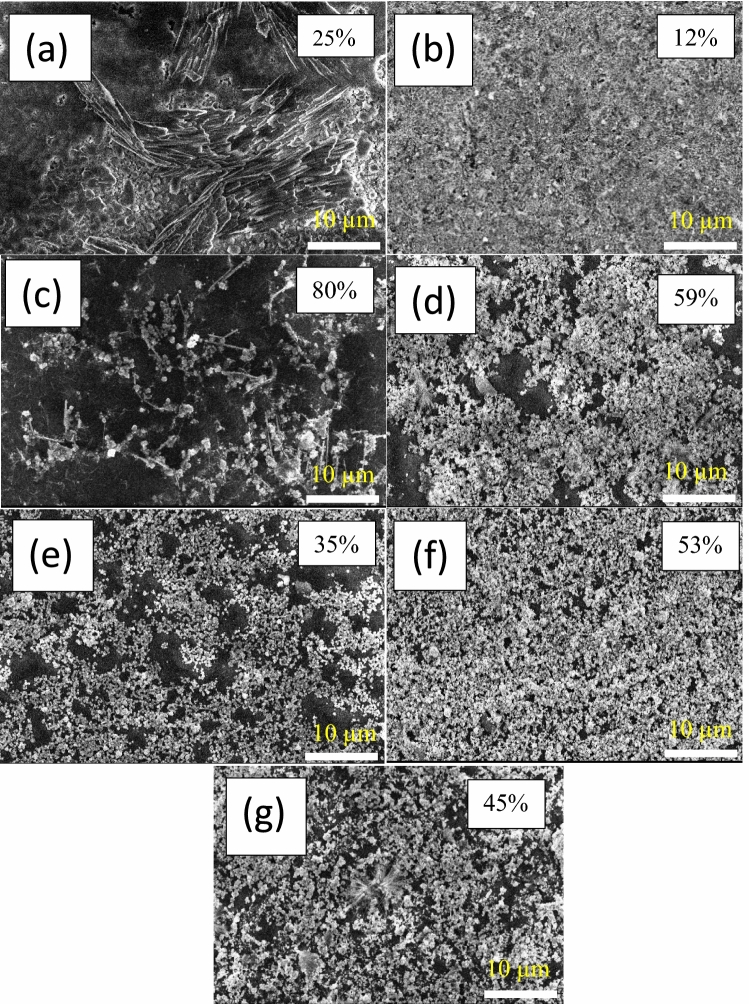
SEM images of AgNWs electrodes with different conditions. (**a**) CuCl_2_ with PVP 40 K, (**b**) CuCl_2_ with PVP-1,3 M, (**c**) CuCl_2_ with mixed PVP (1:1) volume ratio, (**d**) HCl with PVP (1:1) volume ratio, (**e**) HCL with PVP-1,3 M, (**f**) HCl with PVP-40 K, (**g**) CuCl_2_ with mixed PVP (1:1) volume ratio and diethylene glycol as a solvent.

The AgNWs are randomly dispersed over the glass substrate (80 µL), as seen in the SEM images of the electrodes in Fig. 12. The existence of silver nanoparticles cause scattering the incident light and reduces the electrode transmission^53^ as shown in Fig. 7S. The long and thin AgNWs are more suitable for optical properties in the transparent electrodes^56^. On the other hand, silver nanoparticles prepared at 100 °C melted and welded together to fill the gaps of AgNWs^62,63^. Most of fabricated electrodes at different reaction’s conditions have excessive amount of silver nanoparticles, making them unsuitable for photovoltaic applications with high sheet resistance (> 2 MΩ sq^−1^) and low transmissions^64^. Despite AgNWs prepared using CuCl_2_ and PVP-1.3 M (Fig. 12b) have high amount of silver nanoparticles, the sheet resistance exhibits 30 Ω sq^-1^ due to PVP layer was dissolved during the electrode treatment. In the case of using CuCl_2_ and mixed-PVP with a volume ratio of 1:1 of PVP-40 K and PVP-1.3 M (S3), pure silver nanowires prepared with around 10 nm thick PVP remained attached with AgNWs lead to high sheet resistance and high transmission (80%).

**Fig. 13:**
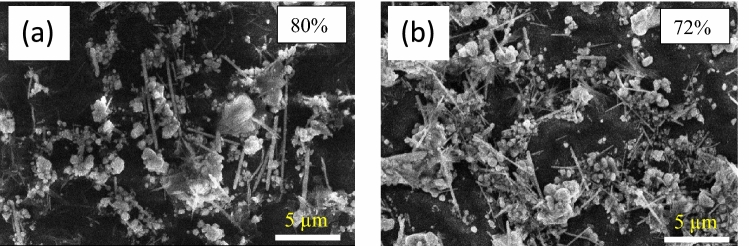
SEM images of AgNWs electrodes synthesized by CuCl_2_ and PVP (1:1) volume ratio, with different coated amounts (**a**) 80 µL (**b**) 200 µL.

The low NW-NW contact between nanowires is the reason for the high sheet resistance. S3 was further investigated using different coating amounts. 80 µL of AgNWs was not sufficient to prepare a transparent electrode, as shown in Fig. 13a as the electrode exhibits high sheet resistance. While increasing the amount of AgNWs to 200 µl (Fig. 13b), the transmission is reduced to 72% and sheet resistance records around 800 KΩ/sq.

## Conclusion

We concluded that mixing two different molecular weights of PVP is essential for synthesizing pure AgNWs (L = 3.7 µl, D = 242 nm, aspect ratio = 15.3). XRD patterns confirmed that the case of high molecular weight of PVP was required to enhance the crystallinity of AgNWs. Moreover, increasing reaction temperature enhanced (111) facet growth in silver atoms, leading to the high yield of AgNWs. Diethylene glycol was not effective in reducing silver cations, and a high amount of silver nitrate accumulated, forming amorphous nanowires. The thickness of PVP employed a crucial role in fabrication of AgNWs electrode. The findings showed that 1 nm of PVP thickness with existing of nanoparticles in the final electrodes can be more suitable than pure nanowires with 10 nm of PVP.

## Supplementary Information

Supplementary Figures.

## Data Availability

All data generated or analysed during this study are included in this published article [and its supplementary information files].
