# Supplementary material for: Preparation of silver nanowires with controlled parameters for conductive transparent electrodes
Source: Sci Rep. 2024 Sep 9;14:20986. doi: 10.1038/s41598-024-70789-6 (PMC11385216; doi:10.1038/s41598-024-70789-6)
Supplement: Supplementary file 1 — Supplementary Figures. [file 41598_2024_70789_MOESM1_ESM.docx]

**Preparation of Silver Nanowires with Controlled Parameters
for Conductive Transparent Electrodes**

**Ahmed Abdel Salam^a^, Shaker Ebrahim^a^, Moataz Soliman^a^, Azza Shokry^a*^**

^a^ Department of Materials Science, Institute of Graduate Studies and Research, Alexandria University, P.O. Box 832, Egypt.

***Corresponding Author***

* **Azza Shokry**. E-mail: azzashokry@alexu.edu.eg Tel: +20 01224788928.

**Keywords:** Silver nanowires; Polyvinylpyrrolidone; Control agent; Polyol; Transparent electrode

**‎Study the Effect of Different Molecular Weights**

**Using Hydrochloric Acid as a Controlling Agent**

**‎**

**Figure 1S.** FT-IR spectra of samples of AgNWs prepared using HCl with different molecular weights of PVP (a) PVP-40K, (b) ‎PVP-1.3M, (c) 1:1 Volume ratio of PVP-40K and PVP-1.3M

**‎Using Copper Chloride as a Controlling Agent‎‎**

**Figure 2S**. FT-IR spectra of samples of AgNWs prepared using CuCl_2_ with different molecular weights of PVP (a) PVP-40K, (b) ‎PVP-‎‎1.3M, (c) 1:1 Volume ratio of PVP-40K and PVP-1.3M.

**The Effect of Synthesizing Temperature**

**Figure 3S.** UV-Vis spectra of AgNWs synthesized using CuCl_2_ and mixed PVP‎ at ‎different temperatures (a) 130 ‎֯‎°C, (b) 150 °C, (c) 170 °C.‎

**Figure 4S.** FT-IR spectra of AgNWs synthesized using CuCl_2_ and mixed PVP
at different temperatures (a) 130 ֯°C, (b) 150 °C, (c) 170 °C**.**

**The Effect of Reducing Agents**

**Figure 5S.** UV-Vis spectra of AgNWs synthesized using CuCl_2_ and mixed PVP with different reducing agents (a) ethylene glycol (b) diethylene glycol


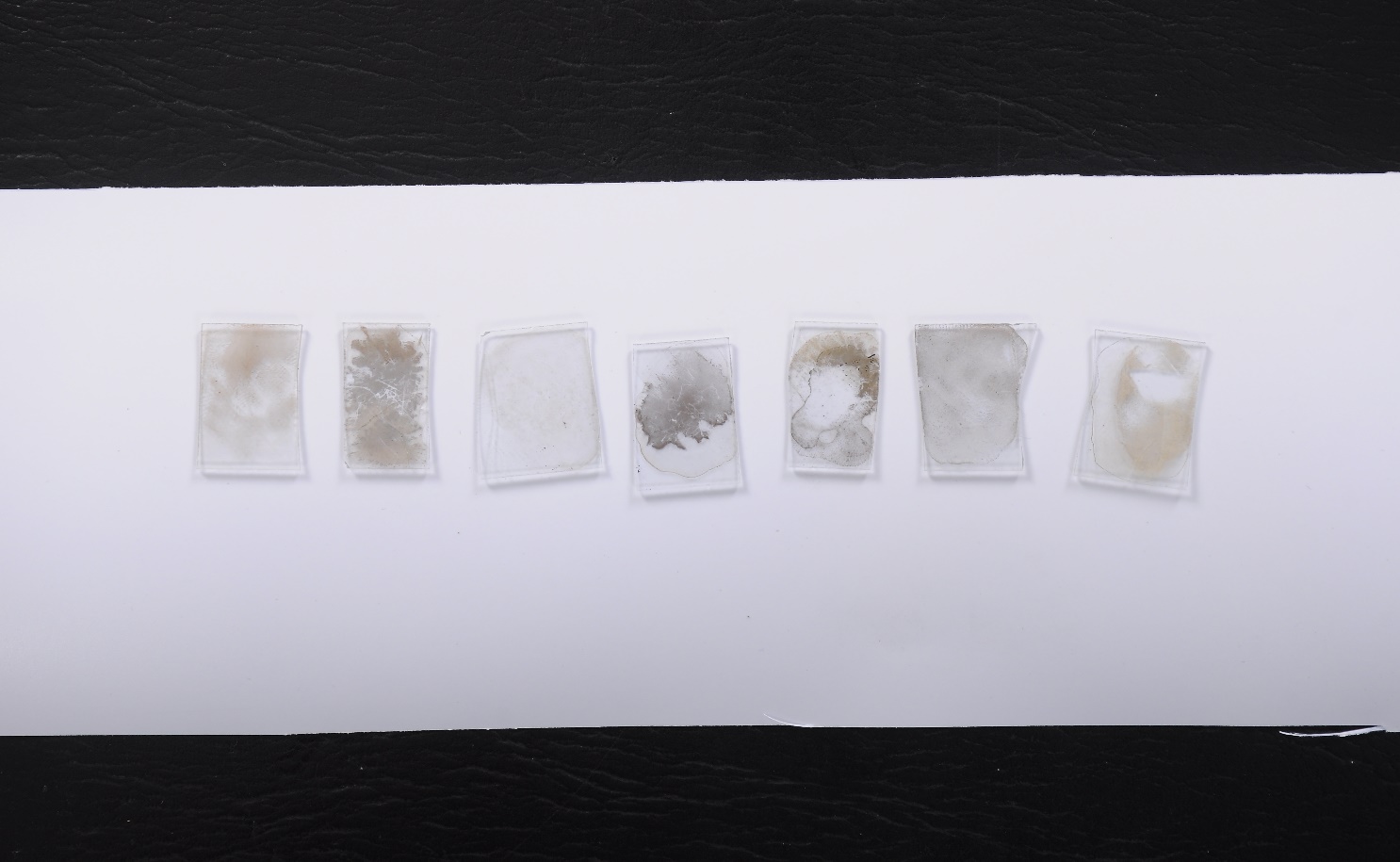
**Preparation of Transparent Conducting Electrode**


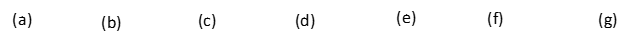


**Figure 6S.** Photographs of AgNWs electrodes synthesized at different conditions (a) CuCl_2_ with PVP 40K, (b) CuCl_2_ with PVP-1,3M, (c) CuCl_2_ with PVP(1:1) volume ratio, (d) HCl with PVP-1,3M, (e) HCL with PVP(1:1) volume ratio, (f) HCl with PVP-40K, (g) CuCl_2_ with PVP (1:1) volume ratio- diethylene glycol


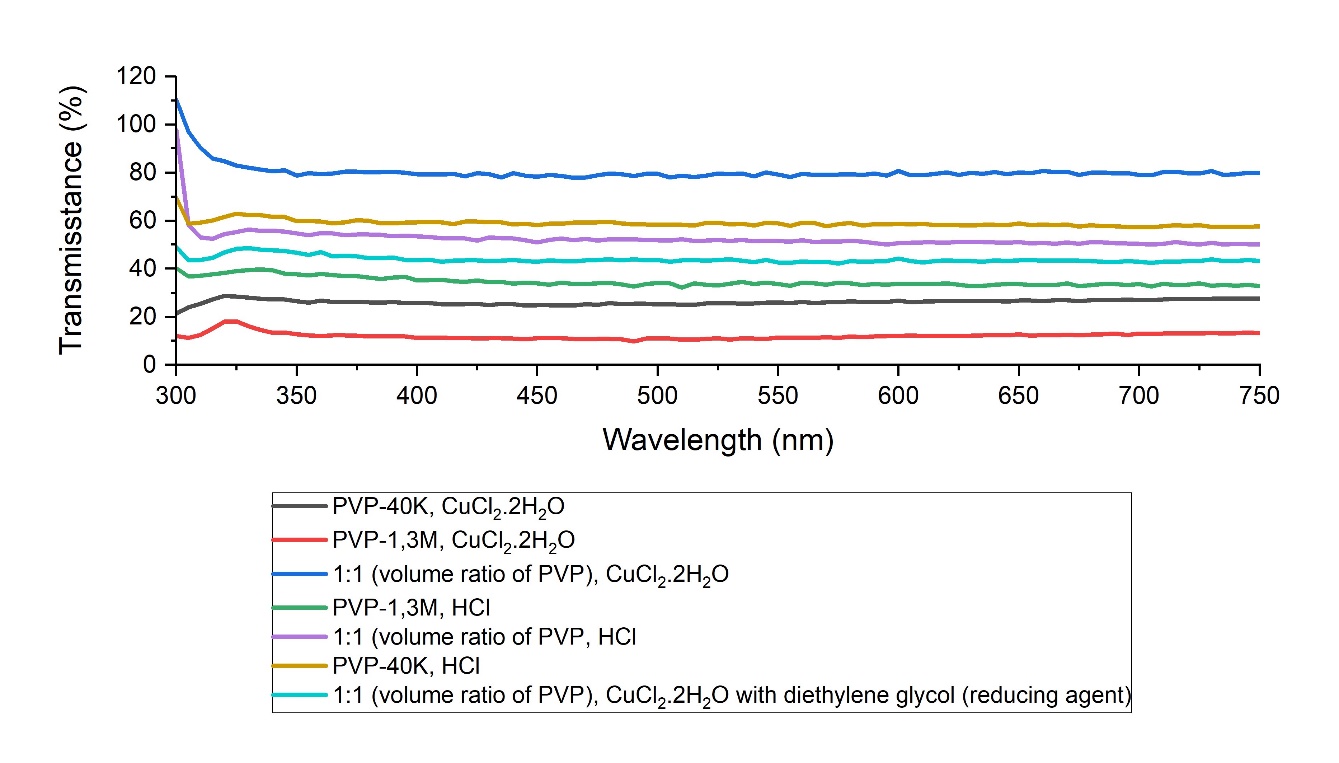


**Figure 7S.** UV-Vis spectra of the transmission of different AgNWs transparent electrodes prepared at different conditions.
